# Physicochemical and Functional Properties of Yeast-Fermented Cabbage

**DOI:** 10.4014/jmb.2302.02025

**Published:** 2023-06-20

**Authors:** Ahhyeon Chun, So Jeong Paik, Jongbeom Park, Ryeongeun Kim, Sujeong Park, Sung Keun Jung, Soo Rin Kim

**Affiliations:** 1 School of Food Science and Biotechnology, Kyungpook National University, Daegu 41566, Republic of Korea; 2 Research Institute of Tailored Food Technology, Kyungpook National University, Daegu 41566, Republic of Korea

**Keywords:** Anti-inflammatory activity, *Saccharomyces cerevisiae*, sulforaphane

## Abstract

Microbial fermentation is often used to improve the functionality of plant-based food materials. Herein, we investigated changes in the physicochemical and functional properties of cabbage during yeast fermentation to develop new products using fermented cabbage. Among the 8 types of food-grade yeast, both *Saccharomyces cerevisiae* and *Saccharomyces boulardii* fermented 10% cabbage powder solution (w/w) the most effectively, leaving no soluble sugars after 12 h of fermentation. In addition, the yeast fermentation of cabbage resulted in functionally positive outcomes in terms of sulforaphane content, antioxidant properties, and anti-inflammatory activity. Specifically, the yeast-fermented cabbages contained about 500% more sulforaphane. The soluble fraction (5 μg/ml) of yeast-fermented cabbage had no cytotoxicity in murine RAW 264.7 cells, and the radical-scavenging capacity was equivalent to 1 μg/ml of ascorbic acid. Moreover, cabbage fermented with *S. boulardii* significantly suppressed both lipopolysaccharides (LPS)-induced nitric oxide production and LPS-induced reactive oxygen species production in RAW 264.7 cells, suggesting a potential anti-inflammatory effect. These results support the idea that yeast fermentation is promising for developing functionally improved cabbage products.

## Introduction

Cabbage (*Brassica oleracea* var. *capitata*) is beneficial for health because it contains large amounts of polyphenols, flavonoids, and glucosinolates [1]. Antioxidant phytochemicals such as polyphenols and flavonoids are recommended for a balanced diet as nutrients that can prevent and repair free radical damage [2]. Glucosinolates and their degraded substances have physiological activity in the human body [3].Glucoraphanin, one of the glucosinolates, is decomposed by myrosinase during the processing of cabbage. Produced glucosinolates can be degraded to sulforaphane, which has pharmacological properties, including antioxidant, anti-inflammatory, and anti-cancer activities [4].

Sauerkraut is a well-known fermented cabbage product made through natural fermentation that relies on native lactic acid bacteria (LAB), which are commonly used as a starter in the production of probiotic foods [5, 6]. Studies have shown that the consumption of probiotics has several health benefits, such as lowering serum cholesterol levels, improving stomach health, and boosting the immune system [7-10]. The antioxidant and anti-inflammatory effects of fermented cabbage using LAB have also been the subject of recent research [11, 12]. However, the number of studies on cabbage fermented with yeast is insignificant.

Yeast is a valuable microorganism mainly used in the industrial fermentation of food [13]. Yeast cell walls contain components with immune-modulatory capabilities, such as beta-glucan and mannan oligosaccharides [14]. Beta-glucans from yeast increase cytokine production [14], and mannan oligosaccharides can alter the immune response [15]. Therefore, yeast is of interest as a potential immune modulator. In this study, we investigated the physicochemical properties of fermented cabbage, found yeast suitable for cabbage fermentation, and confirmed the increase in sulforaphane as well as the antioxidant and anti-inflammatory effects due to fermentation.

Inflammation results from an abnormal immune response to environmental risk factors, such as pathogens, heavy metals, and ambient pollutants [16]. Chronic and abnormal inflammation causes various metabolic diseases, including diabetes, cardiovascular disease, and cancer [17]. Although reactive oxygen species (ROS) and nitric oxide species (NOS) benefit cellular signaling and kill pathogens, abnormal ROS and NOS production results in inflammation, leading to host cell and tissue damage. Many studies have indicated that consuming natural food materials could overcome ROS- and NO-mediated inflammation in vitro and in vivo [18, 19]. As such, developing antioxidative food materials can be seen as a promising strategy for preventing inflammatory metabolic diseases.

## Materials and Methods

### Yeast Strain and Culture Conditions

In this study, 8 types of food-grade yeast strains were used: *Yarrowia lipolytica* (ATCC 18942), *Debaryomyces hansenii* (ATCC 36239), *Saccharomyces boulardii* (ATCC MYA-796), *Kluyveromyces marxianus* (KCTC 17694), and *S. cerevisiae* (EC1118) were purchased from their suppliers, and *Hanseniaspora uvarum* (KCTC 15386BP), *Pichia kluyveri* (KCTC 15385BP), and *Wickerhamomyces anomalus* (KCTC 15387BP) were originally isolated from peaches. A single colony on YPD (1% yeast extract, 2% peptone, and 2% glucose) agar was inoculated in YPD broth and incubated at 30°C and 250 rpm for 24 h to prepare the culture of yeast cells.

### Cabbage Preparation

Cabbage was cut into appropriate sizes, washed, and dried at 60°C for 3 days. The dried cabbage was pulverized to prepare a powder sample and stored at -80°C before the experiment. The moisture content of the cabbage powder was 14.8%, which was measured using the atmospheric pressure drying method at 105°C.

### Fermentation Condition

After making a 10% (w/w) cabbage powder solution using distilled water, we sterilized it at 121°C for 15 min. The precultured yeast cells were inoculated at an initial cell density of 0.5 g DCW/L before being fermented for 24 h at 30°C and 250 rpm.

### pH, Soluble Solids (°Bx), and Viable Cell Counts

A total of 0.5 ml of the sample was placed in a 1.5-ml microtube, and the pH of the cabbage powder was measured before and after fermentation using a pH meter (SevenCompact pH Meter S220, Mettler Toledo, USA). The sample was centrifuged at 15,928 ×*g* for 10 min; 0.3 ml of the supernatant was used; and soluble solids (°Bx) of cabbage powder were measured before and after fermentation using a hand refractometer (Atago Pocket Pal-1, Atago Co. Ltd., Japan). The fermented broth of cabbage powder was serially diluted, spread on YPD agar medium, cultured at 30°C for 24 h, and formed colonies were counted and converted to CFU/ml.

### High-Performance Liquid Chromatography (HPLC) Analysis

The glucose, fructose, glycerol, acetate, and ethanol concentrations of the hydrolysis and fermentation samples were analyzed using HPLC (HPLC 1260 series, Agilent Technologies, USA) equipped with a Rezex-ROA Organic Acid H+ column (8%, 150 mm × 4.6 mm; Phenomenex Inc., USA). The analytes were eluted with 0.005 N H2SO4 at 0.6 ml/min and 50°C, as previously described [20].

Sulforaphane was analyzed using HPLC (Waters, Alliance 2796 Separations System), with a 2996 photodiode array detector (Waters) connected to a Kinetex C18 100A column (5 μm, 250 mm × 4.6 mm: Phenomenex Inc.). The filtrate was analyzed by acetonitrile/water (3:7, v/v) isocratic elution at a flow rate of 0.6 ml/min. A UV/Vis spectrophotometer (Optizen NanoQ, Mecasys Co., Korea) at a wavelength of 205 nm was used to detect sulforaphane.

### Sulforaphane Extraction

The sulforaphane content was determined by modifying the method described in [21]. Cabbage was fermented with *S. boulardii* and freeze-dried to obtain a powder. A total of 5 g of the freeze-dried sample was added to 2.5 g of anhydrous sodium sulfate in 50 ml of dichloromethane, ultrasonically extracted at 30°C for 5 min, and centrifuged to obtain a supernatant. The pellet was rehydrated in 50 ml of dichloromethane, and extraction was performed again. The first and second supernatants were vacuum-filtered and dehydrated at 30°C using a rotary evaporator. The residue was dissolved in 4 ml of acetonitrile and filtered through a 0.2-μm syringe filter.

### Sample Preparation for Functional Analysis

Two yeasts, *S. cerevisiae* (SC) and *S. boulardii* (SB), which are the fastest yeasts fermenting free sugars from cabbage, were selected to analyze the functionality of yeast-fermented cabbage in vitro and at the cellular level. Fermentation was carried out under the same conditions as before, except that the fermentation time was shortened to 12 h, considering that all sugars were consumed within 12 h. The fermented cabbage solution was centrifuged to collect only the supernatant, which was then lyophilized to prepare a sample. As a control, the supernatant of a 10% unfermented cabbage solution was also freeze-dried and used in the same manner. Using the sample, cytotoxicity was evaluated first to set the treatment concentration. Next, the antioxidant effect, immune-enhancement effect using RAW 264.7 cells, and anti-inflammatory effects were confirmed.

### Functional Analysis

**Cell culture**. Mouse macrophage RAW 264.7 cells were purchased from the Korean Cell Line Bank (Seoul, Korea) and maintained in a DMEM/high-glucose medium (Hyclone, USA) supplemented with 10% fetal bovine serum (FBS, Thermo Scientific Hyclone, USA) and 1% penicillin–streptomycin solution (Thermo Scientific HyClone, USA). Cells were grown to 70–80% confluency in a T-75 flask and cultured for 2 days at a density of 2 × 10^5^ cells/ml. Cells were then incubated at 37°C in a humidified 5% CO_2_ incubator (Thermo Fisher Scientific, USA).

**Cell viability.** RAW 264.7 cells were seeded at a density of 3 × 10^5^ cells/ml with 100 μl/well in a 96-well plate. Yeast-fermented cabbages were treated at a concentration of 5 μg/ml for 24 h until the cells reached 70%–80%confluency. A total of 10 μl/well of 3-(4,5-Dimethylthiazol-2-yl)-2,5-Diphenyltetrazolium Bromide (MTT) solution was added to the mixture and incubated for 2 h. Later, 80 μl/well of the mixture was discarded, and 100 μl/well of dimethyl sulfoxide (DMSO) solution was added. After 30 min of incubation in a dark room, cell viability was detected with a microplate reader (Bio-Rad Inc., USA) at an absorbance of 595 nm.

### Free Radical-Scavenging Activity

The free radical-scavenging activities of yeast-fermented cabbage were evaluated using 2,2'-Azino-Bis(3-Ethylbenzothiazoline-6-Sulfonic Acid) (ABTS) and 2,2-diphenyl-1-picryl-hydrazyl-hydrate (DPPH) assays. To test the ABTS radical-scavenging activity, a 5 mg/ml ABTS tablet was diluted in distilled water, and 2.45 mM potassium persulfate was added to make a 7 mM ABTS solution. ABTS radical cations were generated between the ABTS-diluted solution and potassium persulfate by reacting for 12–16 h. The solution containing ABTS radical cations was diluted with sterilized phosphate-buffered saline (PBS) to absorb at 750 nm. A 96-well plate was filled with 100 μl/well yeast-fermented cabbage solution (5 μg/ml) and ascorbic acid at various concentrations diluted in sterilized water. Next, 100 μl/well of diluted ABTS solution was added. Absorbance was detected at a wavelength of 750 nm using a microplate reader (Bio-Rad Inc.) after 30 min in a dark room. A 40 μg/ml DPPH solution was diluted in methanol and transferred to a 96-well plate at a concentration of 100 μl/well to test the DPPH radical scavenging activity. Finally, 100 μl of DPPH solution was added to each well and allowed to react for 30 min in a dark room. Absorbance was detected at a wavelength of 595 nm using the same procedure as with the ABTS.

### NO Production

RAW 264.7 cells were seeded at a density of 3 × 10^5^ cells/ml with 200 μl/well in a 96-well plate. Yeast-fermented cabbages were pretreated at a concentration of 5 μg/ml for 1 h after the cells reached 70%–80% confluency. Cells were then treated with 1 μg/ml of LPS. After incubation for 24 h, 100 μl/well of supernatant was transferred to a new 96-well plate and treated with 100 μl/well of a Griess reagent A and B mixture. NO production was detected with a microplate reader at an absorbance of 550 nm after a reaction of 30 min.

### ROS Production

RAW 264.7 cells were seeded at a density of 3 × 10^5^ cells/ml with 200 μl/well in a 96-well plate. Yeast-fermented cabbages were pretreated at a concentration of 5 μg/ml for 1 h until the cells reached 70%–80% confluency. Next, the samples were treated with 1 μg/ml of LPS. After incubation for 24 h, 200 μl/well of sterilized PBS was used for washing twice. Later, 200 μl/well of 2’,7’-dichlorofluorescein diacetate (DCFH-DA) solution was added. After 30 min of incubation in a dark room, 200 μl/well of sterilized PBS was used for two rounds of washing. ROS production was detected using a fluorescent plate reader (SpectraMax; Molecular Devices Corporation, USA) at an absorbance between 485 nm and 538 nm.

## Results

### Physicochemical and Microbiological Properties of Yeast-Fermented Cabbage

**Fig. 1:**
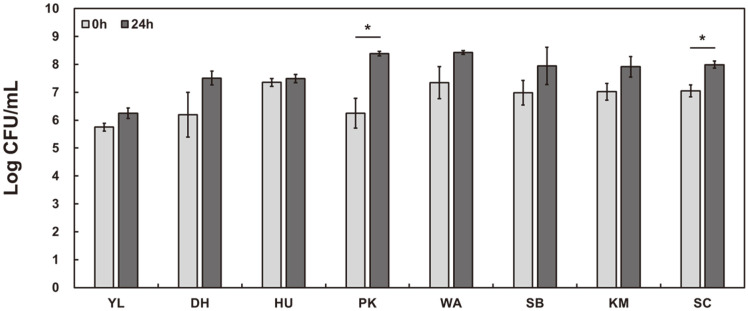
Changes in the viability of yeast cells during fermentation of a 10% (w/w) cabbage powder solution. YL: *Yarrowia lipolytica*; DH: *Debaryomyces hansenii*; HU: *Hanseniaspora uvarum*; PK: *Pichia kluyveri*; WA: *Wickerhamomyces anomalus*; SB: *Saccharomyces boulardii*; KM: *Kluyveromyces marxianus*; SC: *Saccharomyces cerevisiae*.

**Fig. 2:**
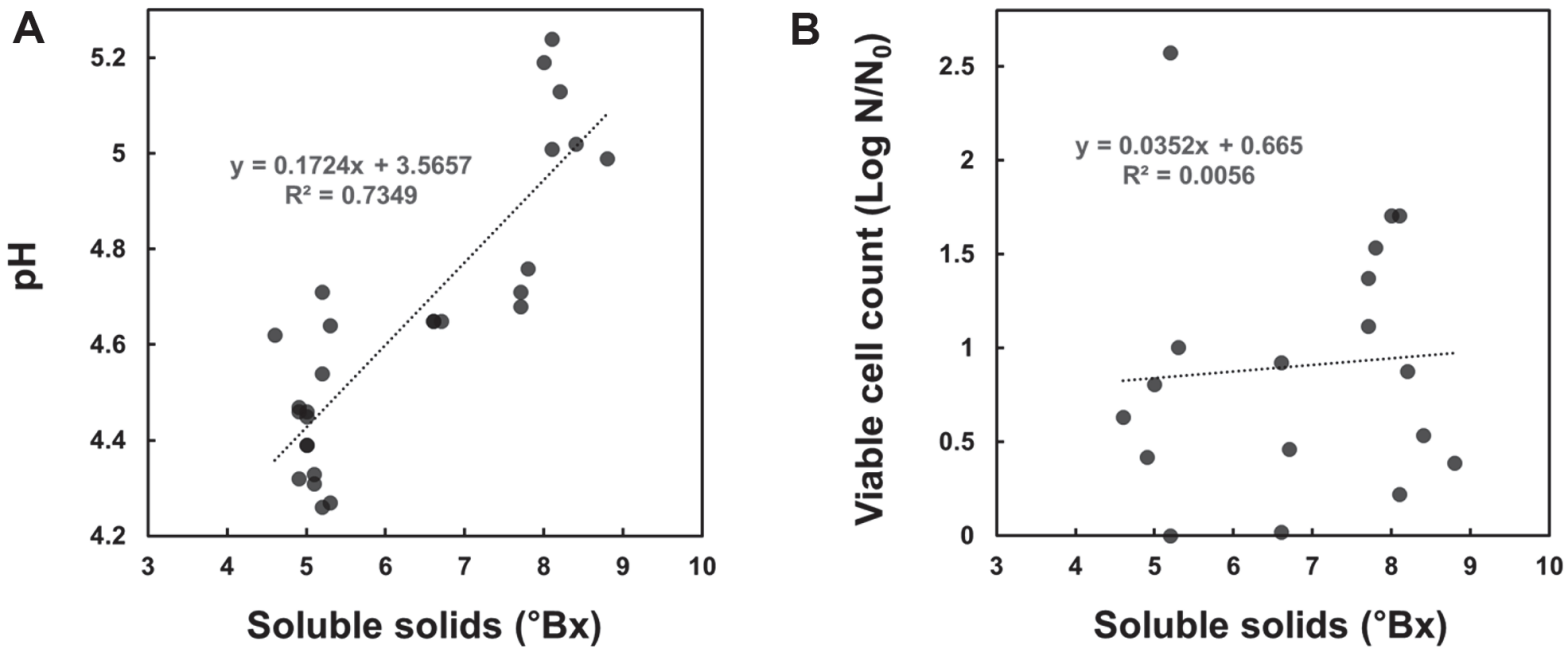
Correlation between solid soluble content (°Bx) and pH (A) or viable cell count (B, log N/N_0_) of fermented cabbage by 8 types of yeast. N: viable cell counts after 24 h of fermentation, N_0_; viable cell counts at 0 h.

Eight types of yeast were inoculated at 10^6^ CFU/ml or more to ferment 10% of the cabbage powder solution. As a result, the viable cell count increased after fermentation in all yeasts, and *P. kluyveri* and *S. cerevisiae* increased significantly (*p* < 0.05) (Fig. 1). After cabbage fermentation, all yeasts except *Y. lipolytica* tended to decrease the soluble solid content and pH (Table 1). The lower the solid soluble content, the lower the pH (Fig. 2A). An increase in viable cells was observed after fermentation (Table 1), but there was no correlation between the degree of increase in viable cells and the degree of decrease in soluble solids (Fig. 2B).

### Fermentation Profiles of Yeast with Cabbage

**Fig. 3:**
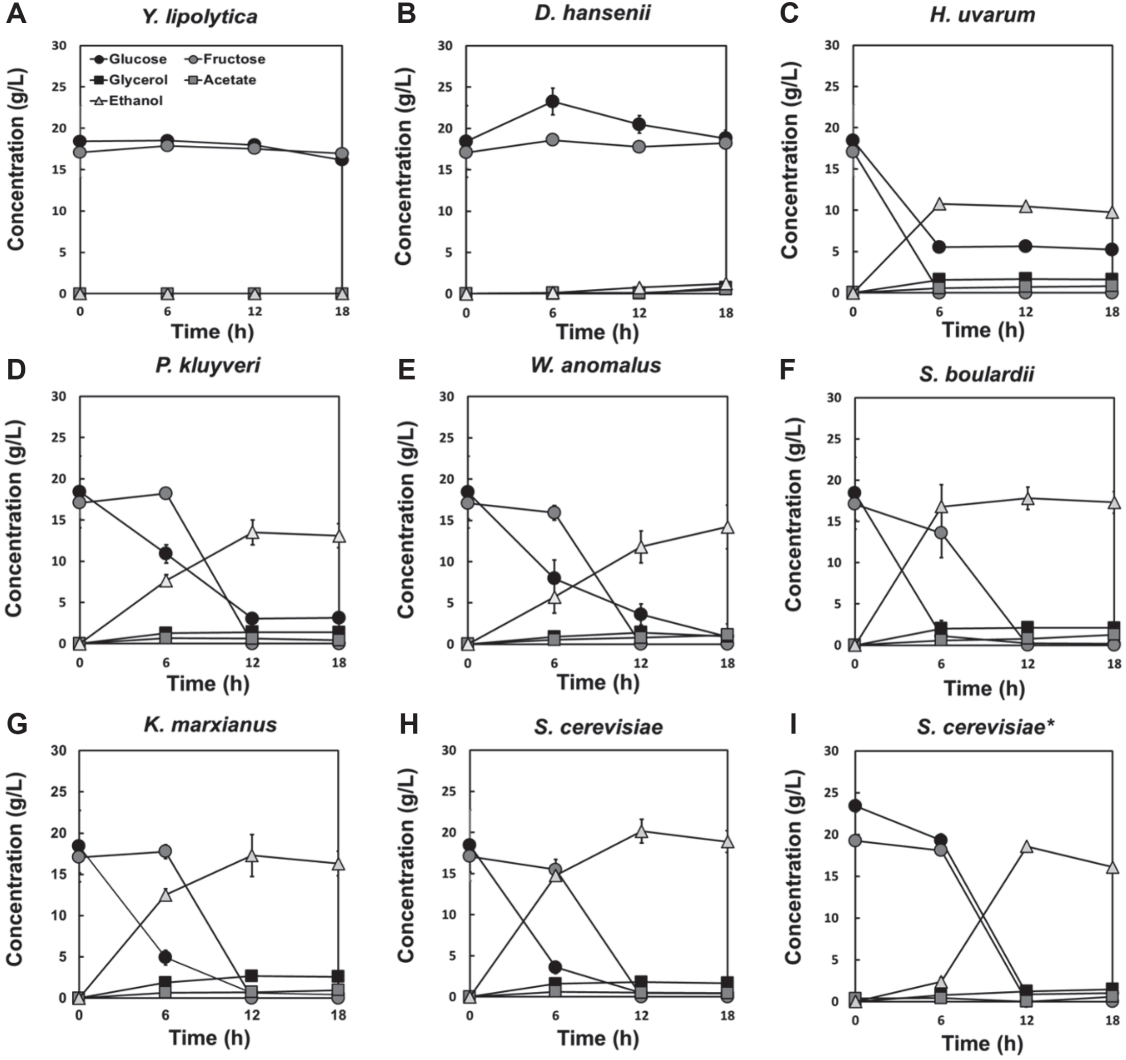
Fermentation profiles of 8 types of yeast after fermenting 10% (w/w) cabbage powder solution. *Y. lipolytica* (**A**) *D. hansenii* (**B**) *H. uvarum* (**C**) *P. kluyveri* (**D**) *W. anomalus* (**E**) *S. boulardii* (**F**) *K. marxianus* (**G**) and *S. cerevisiae* (**H**) were inoculated at an initial cell density of 0.5 g/l (A-H) or 0.05 g/l (**I**). The results at 24 h were excluded because the profiles were mostly consistent after 18 h. All experiments were performed in biological triplicates.

Fermentation was performed to determine the degree of substrate consumption and metabolite production by yeast in a 10% cabbage powder solution. In the cabbage powder, approximately 36% (w/v) free sugars were present as glucose and fructose. *Y. lipolytica* and *D. hansenii* rarely consumed cabbage-free sugars and did not produce metabolites (Fig. 3A and 3B). *H. uvarum* and *P. kluyveri* consumed fructose but not glucose within 12 h. The maximum ethanol yield of *H. uvarum* was approximately 10.8 g/l, and that of *P. kluyveri* was approximately 13.5 g/l (Fig. 3C and 3D). *W. anomalus* consumed all sugars at 18 h of fermentation and produced approximately 14.2 g/l of ethanol (Fig. 3E). *S. boulardii*, *K. marxianus*, and *S. cerevisiae* consumed all sugars within 12 h of fermentation and produced 17.8, 17.3, and 20.1 g/l of ethanol, respectively (Figs. 3F-3H). Additionally, even when fermentation was performed by lowering the concentration of *S. cerevisiae* 10 times, all sugars were consumed within 12 h of fermentation and approximately 18.6 g/l of ethanol was produced (Fig. 3I).

### Functional Components of Fermented Cabbage

The sulforaphane content in cabbage fermented with *S. cerevisiae* and *S. boulardii* was 1.59 ± 0.66 mg/100 g and 1.40 ± 0.41 mg/100 g, respectively (Table 3). This was 5.6–6.4 times higher than the sulforaphane content of 0.25 ± 0.10 mg/100 g of the original cabbage.

### Cell Cytotoxicity of Yeast-Fermented Cabbages and Radical-Scavenging Capacity

**Fig. 4:**
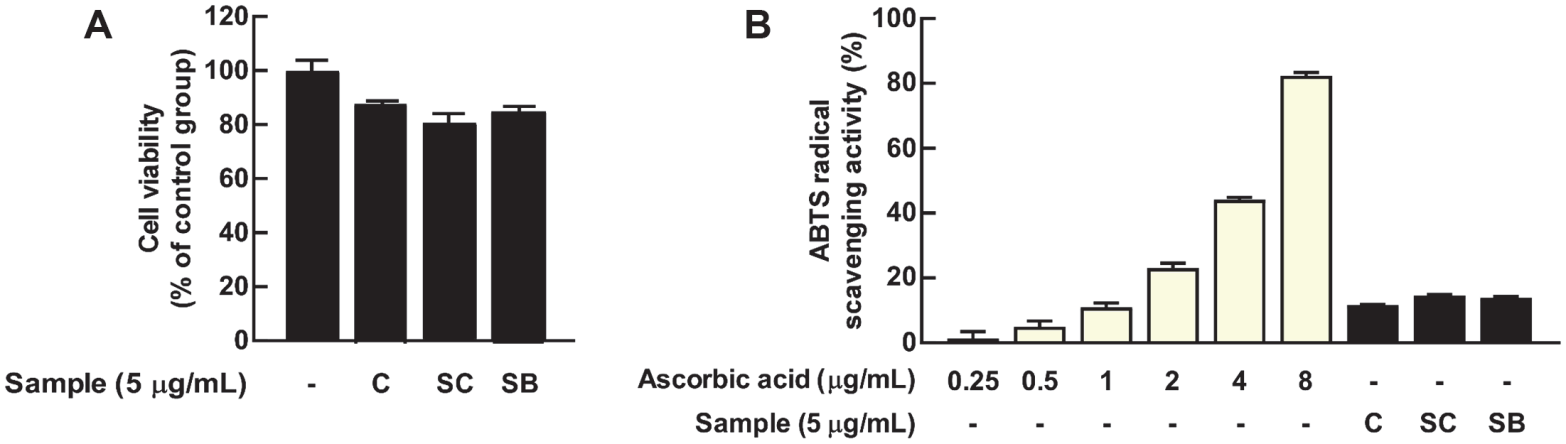
Cell viability of yeast-fermented cabbages in RAW 264.7 cells and the radical scavenging activity. (**A**) Yeast-fermented cabbages have no cytotoxicity at a concentration of 5 μg/ml. The RAW 264.7 cells were treated with 5 μg/ml of fermented cabbages and incubated for 24 h. Cell viability was measured by MTT agents, as described in the Materials and Methods section. (**B**) Yeast-fermented cabbages have ABTS radical-scavenging activity. ABTS radical-scavenging capacity was performed as described in the Materials and Methods section. All data represent the mean ± SD of three independent experiments.

At various concentrations, we evaluated the cytotoxicity of yeast-fermented cabbage on RAW 264.7 cells, and no cytotoxicity was observed at a concentration of 5 μg/ml (Fig. 4A). Three samples of 5 μg/ml slightly reduced cell viability but did not show significant cytotoxicity. Therefore, this concentration was selected for future experiments (Fig. 4A). We next confirmed that yeast-fermented cabbages showed ABTS radical-scavenging activity equivalent to 1 μg/ml of ascorbic acid (Fig. 4B).

### Yeast-Fermented Cabbages Have Antioxidant and Anti-Inflammatory Effects in RAW 264.7 Cells

**Fig. 5:**
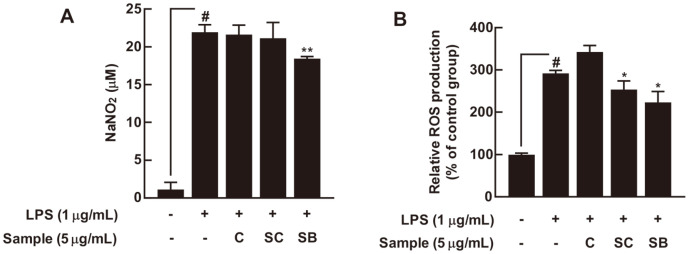
The effects of yeast-fermented cabbage on LPS-induced NO and ROS production in RAW 264.7 cells. (**A**) Cabbage fermented with *S. boulardii* suppressed LPS-induced NO production in RAW 264.7 cells. Cells were pretreated with 5 μg/ml of yeast-fermented cabbage for 1 h before receiving 1 μg/ml of LPS treatment. NO production was measured using Griess reagent, as described in Materials and Methods. (**B**) Cabbage fermented with *S. boulardii* and *S. cerevisiae* significantly suppressed LPS-induced ROS production in RAW 264.7 cells. Cells were pretreated with 5 μg/ml of yeast-fermented cabbages for 1 h before receiving 1 μg/ml of LPS treatment; then, cells were incubated with 20 μM of 2’,7’-dichlorofluorescein diacetate for 30 min. # *p* < 0.05, compared with the control group; * *p* < 0.05 and ** *p* < 0.01, compared with the group exposed to LPS alone.

Because NO, a proinflammatory mediator, and ROS have critical roles in the pathogenesis of inflammation [22], we investigated the effects of yeast-fermented cabbages on LPS-induced NO and ROS production in RAW 264.7 cells. Only cabbage fermented with *S. boulardii* (5 μg/ml) significantly suppressed LPS-induced NO production in RAW 264.7 cells. In contrast, cabbage extract fermented with *S. cerevisiae* had no effect (Fig. 5A). Both test samples of cabbage fermented with *S. cerevisiae* and *S. boulardii* significantly prevented LPS-induced ROS production in RAW 264.7 cells. However, cabbage extract had no effect (Fig. 5B).

## Discussion

Eight types of yeast were inoculated into a 10% cabbage powder solution, and fermentation was performed. As a result, viable cell counts increased in all yeasts. Therefore, it was confirmed that fermentation in cabbage powder is possible only with hydrothermal treatment. Because the solid soluble content decreased in all yeasts after the fermentation of cabbage, we observed that the yeast effectively used the soluble solids of cabbage for fermentation. During fermentation, yeast is known to produce organic acids [23], which decreased pH.

Fermentation in 10% cabbage powder solution was conducted to confirm the substrate consumption and metabolite production level of yeast. *W. anomalus*, *S. boulardii*, *K. marxianus*, and *S. cerevisiae* fermented the soluble sugars effectively. Among these, *S. boulardii* and *S. cerevisiae* are mainly used in the food industry [24, 25]. Therefore, functional evaluation was performed on cabbage samples fermented with *S. cerevisiae* and *S. boulardii*.

A previous study reported that the sulforaphane content of raw broccoli puree after maceration increased by more than 1.9 times when fermented with *Lactobacillus plantarum* [26]. In addition, studies have shown that cabbage fermentation using LAB can help convert glucoraphanin to sulforaphane and increase the content of sulforaphane after fermentation [26]. The change in sulforaphane content in this study is also thought to be due to the conversion of glucoraphanin to sulforaphane during cabbage fermentation. However, future studies will require an analysis of glucoraphanin to support this opinion. Appropriate gradation of drying medium temperature and vacuum drying can improve the formation and retention of sulforaphane [27]. Furthermore, high hydrostatic pressure is nonthermal processing and can effectively increase the sulforaphane content of raw cabbage [28]. A high sulforaphane content will be confirmed in future experiments if these conditions are met.

Once pathogens disrupt mammalian homeostasis in the body, the immune system recruits immune cells such as macrophages and amplifies the production of NO and ROS to remove the foreign intruder [29]. This series of reactions ultimately leads to inflammation. Cardiovascular diseases, diabetes, and cancer are caused by chronic inflammation. Therefore, preventing or controlling inflammation through the regulation of NO and ROS will be one of the strategies to prevent the development of metabolic diseases. Our evaluation of the antioxidant effects of the materials showed that fermented cabbages with *S. cerevisiae* and *S. boulardii* have an antioxidant effect on RAW 264.7 cells. Additionally, all fermented cabbages exhibited ABTS radical-scavenging activity corresponding to 1 μg/ml of ascorbic acid. In the case of extracts, 100 μg/ml is generally used as the experimental extract concentration. In this study, we adopted a 5 μg/ml concentration of samples, which was a relatively low concentration compared to other studies [30, 31].

Abnormal NO and ROS production are closely related to the development of inflammation. Only cabbage fermented with *S. boulardii* suppressed LPS-induced NO production in RAW 264.7 cells. Interestingly, cabbage fermented with both *S. cerevisiae* and *S. boulardii* suppressed LPS-induced ROS production in RAW 264.7 cells. Because cabbage fermented with *S. boulardii* inhibits LPS-induced NO and ROS production, it seems to be a promising nutraceutical material for fighting off inflammation. NO is produced through iNOS, which is regulated by NF-κB signaling pathways. Therefore, in the future, it will be necessary to evaluate whether these materials affect iNOS expression by regulating the NF-κB signaling network.

## Figures and Tables

**Table 1:** Physicochemical and microbiological properties of yeast-fermented cabbage.

	Soluble solids (°Bx)	pH	Log N/N_0_^1)^
Cabbage solution^2)^	8.43 ± 0.35^a^	5.01 ± 0.02^a^	-
*Yarrowia lipolytica*	8.10 ± 0.10^b, a^	5.19 ± 0.06^b^	0.50
*Debaryomyces hansenii*	7.73 ± 0.06^b^	4.72 ± 0.04^c^	1.32
*Hanseniaspora uvarum*	6.63 ± 0.06^c^	4.65 ± 0.00^c, d^	0.14
*Pichia kluyveri*	5.23 ± 0.06^d^	4.63 ± 0.09^c, d^	2.14
*Wickerhamomyces anomalus*	5.13 ± 0.21^d^	4.28 ± 0.03^e^	1.08
*Saccharomyces boulardii*	5.07 ± 0.06^d^	4.34 ± 0.04^e, f^	0.96
*Kluyveromyces marxianus*	4.97 ± 0.06^d^	4.44 ± 0.04^f, g^	0.90
*Saccharomyces cerevisiae*	4.83 ± 0.21^d^	4.51 ± 0.10^d, g^	0.94

^1)^Changes in viable cell counts. N: viable cell counts after 24 h fermentation, N_0_; viable cell counts at 0 h.

^2)^10%, w/w.

**Table 2:** Metabolite profiles of cabbage fermented for 24 h using 8 types of yeast.

	Glucose (g/l)	Fructose (g/l)	Glycerol (g/l)	Acetate (g/l)	Ethanol (g/l)
*Yarrowia lipolytica*	15.73 ± 0.38	17.00 ± 0.04	-	-	-
*Debaryomyces hansenii*	16.44 ± 1.15	17.42 ± 0.08	0.68 ± 0.02	0.40 ± 0.03	1.78 ± 0.19
*Hanseniaspora uvarum*	5.22 ± 0.10	-	1.62 ± 0.07	0.88 ± 0.06	9.19 ± 0.10
*Pichia kluyveri*	2.91 ± 0.39	-	0.93 ± 0.14	-	10.35 ± 1.52
*Wickerhamomyces anomalus*	0.42 ± 0.73	-	0.41 ± 0.42	1.35 ± 0.16	12.60 ± 1.98
*Saccharomyces boulardii*	-	-	1.96 ± 0.08	1.46 ± 0.21	15.75 ± 1.77
*Kluyveromyces marxianus*	0.37 ± 0.05	-	2.29 ± 0.07	1.03 ± 0.16	15.21 ± 1.46
*Saccharomyces cerevisiae*	0.29 ± 0.12	-	2.04 ± 1.04	1.10 ± 1.50	17.82 ± 1.96

**Table 3:** Functional components of fermented cabbage.

	Sulforaphane content	Reference
Cabbage (control)	0.25 ± 0.10 mg/100 g powder	This study
Fermented by *S. cerevisiae*	1.59 ± 0.66 mg/100 g powder	This study
Fermented by *S. boulardii*	1.40 ± 0.41 mg/100 g powder	This study
Fresh cabbage	4.1–13.9 mg/100 g dry basis	[27]
Hot air-dried cabbage	7.7–16.7 mg/100 g dry basis	[27]
Pressurized cabbage	0.056 μg/100 g fresh weight	[28]
Raw broccoli	0.48 μg/100 g dry weight	[26]
Fermented broccoli by *L. plantarum*	0.91 μg/100 g dry weight	[26]

S., *Saccharomyces*
